# High-dimensional genomic data bias correction and data integration using MANCIE

**DOI:** 10.1038/ncomms11305

**Published:** 2016-04-13

**Authors:** Chongzhi Zang, Tao Wang, Ke Deng, Bo Li, Sheng'en Hu, Qian Qin, Tengfei Xiao, Shihua Zhang, Clifford A. Meyer, Housheng Hansen He, Myles Brown, Jun S. Liu, Yang Xie, X. Shirley Liu

**Affiliations:** 1 Department of Biostatistics and Computational Biology, Dana-Farber Cancer Institute and Harvard T.H. Chan School of Public Health, Boston, Massachusetts 02215, USA; 2 Center for Functional Cancer Epigenetics, Dana-Farber Cancer Institute, Boston, Massachusetts 02215, USA; 3 Quantitative Biomedical Research Center, Department of Clinical Sciences, University of Texas Southwestern Medical Center, Dallas, Texas 75390, USA; 4 Center for the Genetics of Host Defense, University of Texas Southwestern Medical Center, Dallas, Texas 75390, USA; 5 Center for Statistical Science, Tsinghua University, Beijing 100084, China; 6 Department of Statistics, Harvard University, Cambridge, Massachusetts 02138, USA; 7 Department of Bioinformatics, School of Life Sciences, Tongji University, Shanghai 200092, China; 8 Department of Medical Oncology, Dana-Farber Cancer Institute and Harvard Medical School, Boston, Massachusetts 02215, USA; 9 National Center for Mathematics and Interdisciplinary Sciences, Academy of Mathematics and Systems Science, Chinese Academy of Sciences, Beijing 100190, China; 10 Department of Medical Biophysics, University of Toronto, Toronto, Ontatio M5G 1L7, Canada; 11 Department of Bioinformatics, University of Texas Southwestern Medical Center, Dallas, Texas 75390, USA; 12 Simons Comprehensive Cancer Center, University of Texas Southwestern Medical Center, Dallas, Texas 75390, USA

## Abstract

High-dimensional genomic data analysis is challenging due to noises and biases in high-throughput experiments. We present a computational method matrix analysis and normalization by concordant information enhancement (MANCIE) for bias correction and data integration of distinct genomic profiles on the same samples. MANCIE uses a Bayesian-supported principal component analysis-based approach to adjust the data so as to achieve better consistency between sample-wise distances in the different profiles. MANCIE can improve tissue-specific clustering in ENCODE data, prognostic prediction in Molecular Taxonomy of Breast Cancer International Consortium and The Cancer Genome Atlas data, copy number and expression agreement in Cancer Cell Line Encyclopedia data, and has broad applications in cross-platform, high-dimensional data integration.

## Abstract (web summary)

Analyses of data from high-throughput genomic technologies are challenging given large data dimensionality. Here, Liu and colleagues describe a method called MANCIE (Matrix Analysis and Normalization by Concordant Information Enhancement) that can conduct genomic data normalization and bias correction to detect biologically relevant information.

## Graphical abstract

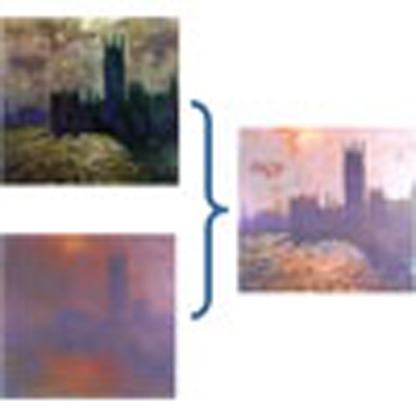

High-throughput genomic technologies have made it possible to generate massive data for studying biological mechanisms or disease aetiology. Such high-dimensional genomic data usually can be presented as a matrix, with each column representing a sample (for example, a patient, a cell type, an experimental condition and so on), and each row representing a genomic feature (for example, a gene, a genomic locus and so on). By computational analyses of these high-dimensional data matrices using dimension reduction (for example, principal component analysis, PCA) or clustering approaches, one can learn characteristic information within samples and identify key features between samples to interrogate biological functions. In many cases, there can be multiple platforms of experiments on the same set of samples and they can generate more than one data matrices. For example, the ENCODE (Encyclopedia of DNA Elements) Consortium generated high-throughput data including ChIP-seq, DNase-seq, and exon array transcriptomes and so on. on a designated panel of human cell lines^1^; The Cancer Genome Atlas (TCGA) program^2^ and the Molecular Taxonomy of Breast Cancer International Consortium (METABRIC)^3^ generated mutation and gene-expression profiles of patient tumours; and the Cancer Cell Line Encyclopedia (CCLE) project^4^ provided copy number, gene expression for over a thousand cancer cell lines. Integrative analysis is critical for obtaining biological insights from these data sets, within which a common challenge exists in identifying and correcting hidden biases in such high-dimensional data matrices.

In high-throughput data with different experimental platforms, it is not uncommon for a subset of samples in a data matrix on one experimental platform to have technical biases^5^,^6^. For example, in a cohort of dozens of samples, the expression and ChIP-seq profiling were conducted under various batches, each with unique biases from sample collection and preparation, array hybridization, sequencing GC content^7^ or coverage differences that are challenging to identify and remove. There have been methods developed to remove batch effect within one data matrix of the same platform. For example, PCA have been used to solve such problems. As an extension of PCA, Sparse PCA^5^ uses the linear combination of a small subset of variables instead of all to generate the principal components and still explains most variances present in the data, while making the dimension reduction and bias removal clearer and easier to interpret^8^. Surrogate variable analysis (SVA)^9^ models the gene-expression heterogeneity bias as ‘surrogate variables' and separate them from primary variables that capture biologically meaningful information. These methods aim to normalize data within the same data matrix from the same platform. However, to our knowledge, methods that can normalize data from different matrices and borrow information between different platforms are still lacking.

Recently, Wang *et al*.^10^ propose similar network fusion (SNF), a method that first generates sample networks from each data platform separately, then uses network fusion to merge the platform-specific networks together with confidence weighting. SNF demonstrated good performance on separating TCGA glioblastoma samples into subtypes using transcriptome and DNA methylome profiles. However, SNF does not provide the normalized data matrices that could be useful in the downstream analysis. In addition, SNF is based on network construction, which could be sensitive to strong biases in a subset of samples that result in ‘high-weight' edges in the network and are difficult to remove in the fusion step. In other words, if the networks generated from each data matrices were too dissimilar, it is difficult to ‘fuse'. A more general applicable method is needed to simultaneously provide better sample clustering and generate normalized data matrices.

To overcome the above challenges, we propose MANCIE (matrix analysis and normalization by concordant information enhancement), an integrative computational method that can conduct data normalization and bias correction by borrowing information from a column-matched associated data matrix. Applied to ENCODE, METABRIC, TCGA and CCLE data, MANCIE showed effectiveness in improved identification of biologically meaningful patterns.

## Results

### Method overview

**Figure 1:**
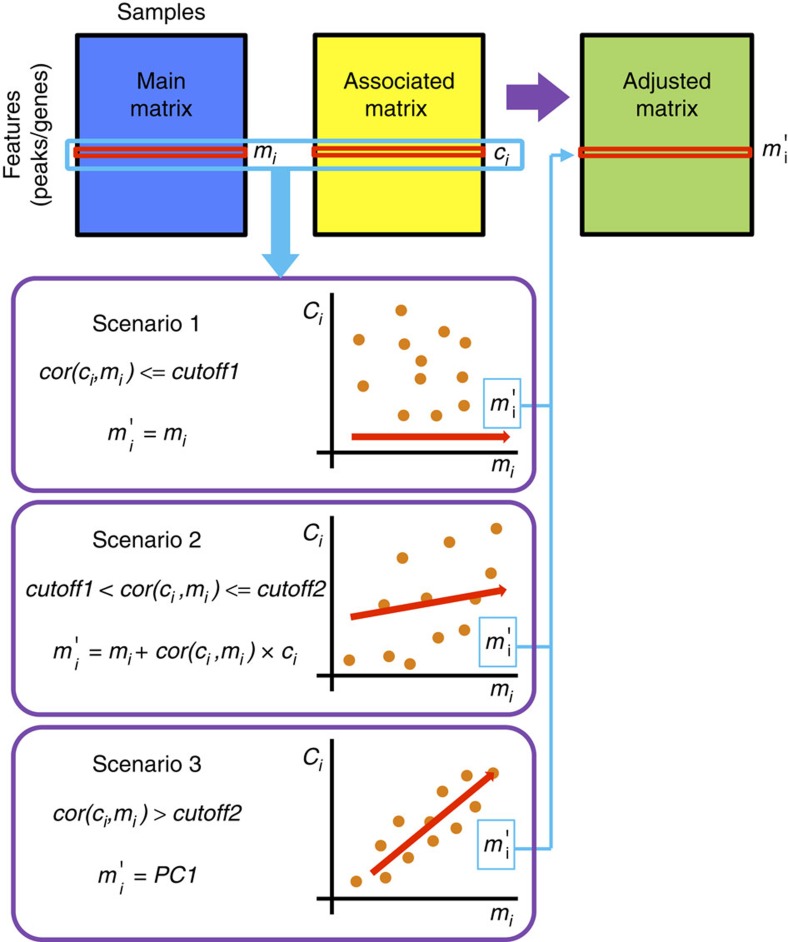
Overview of MANCIE. Each row vector in the adjusted matrix is generated from the corresponding row vectors in the main matrix and the associated matrix. On the basis of the correlation between the main row vector *m*_*i*_ and the associated row vector *c*_*i*_, one of three scenarios will be chosen. See more details in the online methods.

MANCIE takes in two data matrices and adjusts one (thereafter defined as the ‘main matrix') using the other (thereafter defined as the ‘associated matrix') by identifying and maintaining the concordant information and reducing the discordant information between them. The two data matrices contain profiles on the same set of samples generated using different experimental platforms (for example, copy number variation (CNV) and RNA-seq on the same collection of tumours), or generated independently (for example, expression profiles measured at different institutions on the same collection of cell lines). If the rows of the two matrices are unmatched (for example, genes versus ChIP-seq peaks), MANCIE first generates a summarized associated matrix that has matched rows with the main matrix using a biologically motivated matching process (Supplementary Fig. 1, see Methods for details). This matching step requires additional biological information to connect the rows between the two matrices, for example, each gene (as a row vector in the main matrix) will corresponds to a row vector summarized from a few nearby transcription factor (TF) -binding sites (as a few rows in the associated matrix). MANCIE assumes that pairwise sample distance as measured by different platforms should be similar, and discordance in the pairwise distance largely arise from technical biases and/or noises. Therefore, the second and key step of MANCIE adjusts the main matrix row by row by borrowing information from data in the associated matrix. In each row, depending on whether the correlation between the main and associated data is high, moderate or low, MANCIE takes the first principle component (scenario 3), a correlation-weighted sum (scenario 2) or the original data (scenario 1) as the adjusted data, respectively (Fig. 1, see Methods for details). The correlation cutoffs are determined empirically, by making roughly 1/3 of the rows be adjusted under scenario 3. Finally, the output of MANCIE is the normalized adjusted matrix that has the same dimension as the main matrix yet with the information from the associated matrix incorporated. It is worth noting that one can swap the main and associated matrices, so the quality of both data can be improved from each other. We show that this approach is an appropriate approximation of the full Bayesian inference for reducing noises from such data sets (Supplementary Notes). In the following sections, we applied MANCIE on a few data sets generated by large consortia including ENCODE^1^, METABRIC^3^, TCGA^2^ and CCLE^4^ to demonstrate its utility in genomic data integration.

### ENCODE data

**Figure 2:**
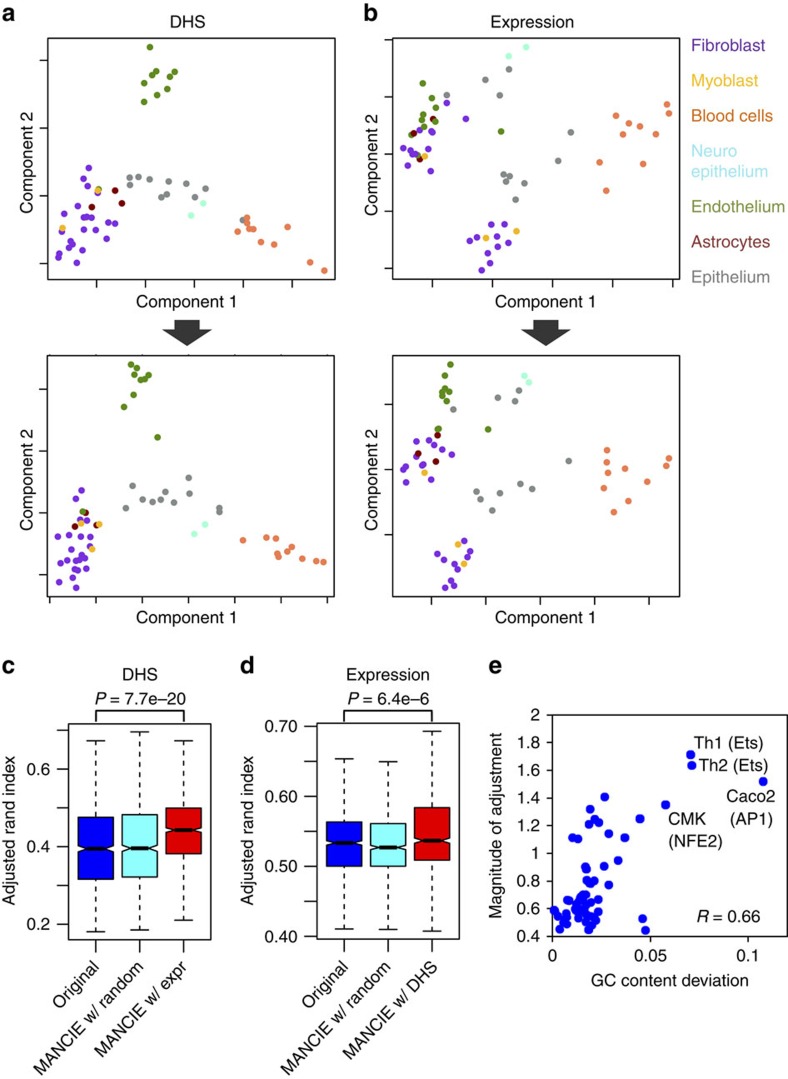
Case study on ENCODE data. (**a**,**b**) Multi-dimensional scaling map representing genomic data from 61 cell lines. Each data point represents a cell line, with its tissue type labelled in the same colour as in the legend. (**a**, top) Raw DHS data; bottom, MANCIE-adjusted DHS data; (**b**, top) Raw expression data; bottom, MANCIE-adjusted expression data. (**c**,**d**) Adjusted Rand index comparing K-means clustering on the data with actual tissue-type clustering. K-means clustering was performed 1,000 times with random seeds. The three boxes represent original data (blue), MANCIE-adjusted with random data matrices (cyan) and MANCIE-adjusted with the other data type (red). (**c**) DHS data, (**d**) gene-expression data. *P* value was calculated using Wilcoxon rank sum test. (**e**) Relationship between the magnitude of MANCIE adjustment and the deviation of GC-content distribution of DNase-seq reads. The magnitude of MANCIE adjustment was calculated as the Euclidean distance between the sample data vectors before and after MANCIE adjustment. The deviation refers to the distance from each sample's data point to the centre of mass in the mean—coefficient of variation map of the GC-content distribution in Supplementary Fig 2c. Labels in the parentheses are the top sequence motif enriched in the most increased DHS in the corresponding cell line after MANCIE adjustment.

From ENCODE consortium^1^, we obtained data from 61 cell lines where both DNase-seq data for chromatin accessibility profiling and Affymetrix exon array data for gene-expression profiling are available. These cell lines can be classified into seven groups by their tissues of origin (Fig. 2). DNase hypersensitive sites (DHS, measured by DNase-seq) mark open chromatin regions that can be considered as a repertoire of all putative *cis*-regulatory elements in the genome^11^,^12^ that regulate gene expression as measured by exon arrays. Although active gene promoters are usually DHS, most DHS are located in introns or intergenic regions, marking distal enhancers^1^,^13^ which are often more dynamic across different cell types or conditions^14^,^15^,^16^,^17^. Focusing on enhancers, we generated the DHS data matrix based on a union set of intronic and intergenic DHS peaks identified from all the DNase-seq data, and obtained the gene-expression data matrix based on the exon array data. We used MANCIE to adjust each data matrix using the other as the associated matrix. Since the rows between the two data matrices are not matched, we generated the summarized associated matrices using the genomic location information of genes (based on the transcription start site, TSS) and DHS (based on the DHS peak centre). For summarization of DHS data around genes, we used up to 50 nearby DHS located within 100 kb from the TSS of each gene as the local sub-matrix for that gene. For summarization of gene-expression data around DHS, we used a similar approach but up to 20 nearby genes, considering that there are much fewer genes than DHS. The adjusted main matrix was then generated by integrating the main matrix with the summarized associated matrix. We conducted multi-dimensional scaling on the adjusted data as well as the raw data, and plotted two principal components with each data point representing a cell line (Fig. 2a,b). We hypothesized that cell lines belonging to the same tissue type should be more similar to each other than cell lines from different tissue types, a pattern previously reported for these data types^16^. Indeed, although MANCIE only aims to make cell lines similar in one platform more similar in the other platform, the end result is that the adjusted data, both DHS and expression data, show better cell line clustering according to their tissue types (Fig. 2a,b). To assess the better clustering quantitatively, we performed K-means clustering with randomly sampled seeds for 1,000 times on each data set and calculated the adjusted Rand index^18^ that measures the similarity between the K-means clustering and the actual tissue-type clustering for each random sample. The average adjusted Rand index is significantly higher for MANCIE-adjusted data than that for the raw data, indicating that MANCIE improves the tissue-type clustering (Fig. 2c,d). In contrast, cell line clustering using SVA-adjusted data is actually worse than using the raw data (Supplementary Fig. 2a,b).

We next investigated the implication of the MANCIE adjustment on the ENCODE data. As GC-content bias is one major sources of biases in next-generation sequencing data, we first checked whether MANCIE can reduce the GC-content biases in the DNase-seq data. For each cell line, we calculated the distribution of the GC-content of all sequence reads in the DNase-seq data set as well as the magnitude of MANCIE adjustment, measured by the Euclidean distance between the corresponding column vectors in the raw and the MANCIE-adjusted data matrices. Cell lines showing GC-content patterns that were farther away from average of all cell lines (Supplementary Fig. 2c) underwent a greater magnitude of MANCIE adjustment than the other cell lines (Fig. 2e and Supplementary Fig. 2d). This result indicates that MANCIE successfully corrected the GC-content biases in the DNase-seq data.

To further evaluate MANCIE performance in adjusting the DNase-seq data, we selected the top 2,000 DHSs with greatest increase after MANCIE adjustment in the cell lines with the biggest adjustment, and performed sequence motif analysis on these DHSs. We found that the sequence motifs enriched in these DHSs usually match cell-type-specific TFs (Fig. 2e). For example, ETS motif is enriched in both TH1 and TH2 cell lines, and the ETS-family TFs ERM and PU.1 are specific to TH1 and TH2 cell lines, respectively^19^,^20^. The motif of megakaryocyte-specific TF NF-E2^17^ is enriched in the megakaryocyte cell line CMK. These results demonstrated that integrated with gene-expression data, MANCIE-adjusted DHSs show an increased pattern of cell-type specificity that is better correlated with cell-type-specific gene-expression pattern. Taken together, MANCIE is able to integrate the genomic DHS data with gene expression and to reduce potential biases, as well as to emphasize biologically meaningful signals.

### METABRIC and TCGA data

**Figure 3:**
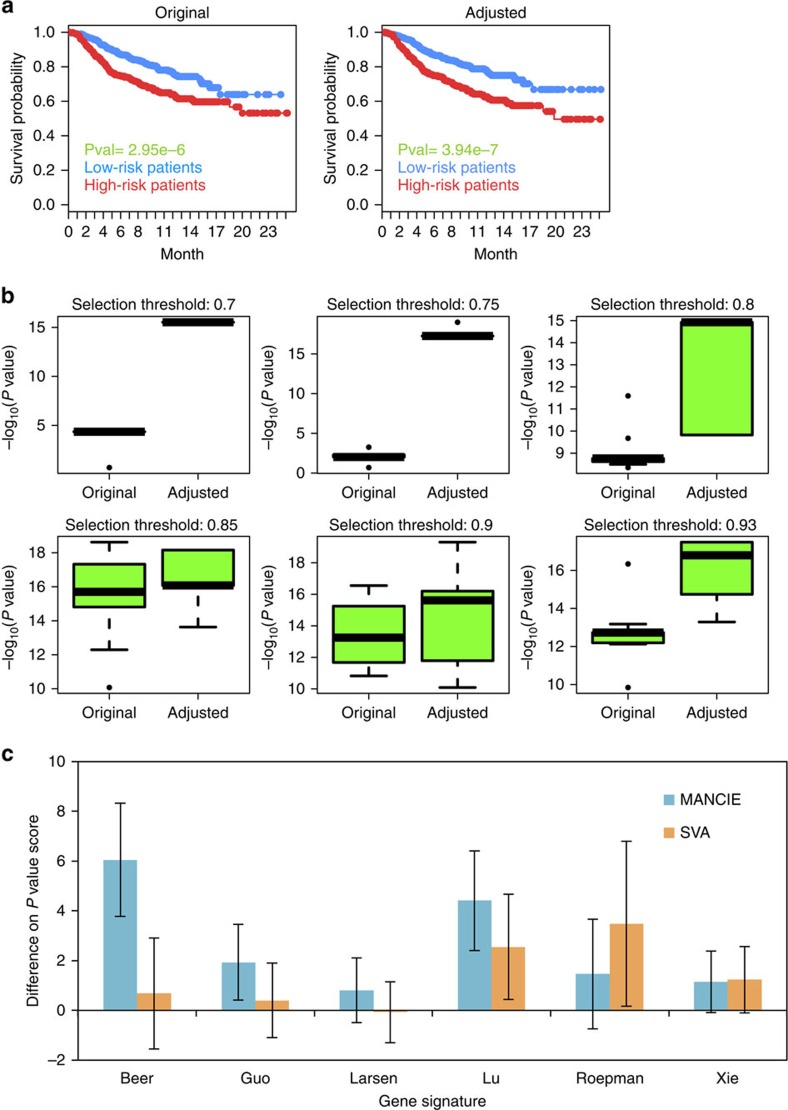
Case studies on METABRIC and TCGA data. (**a**) The Kaplan–Meier plots for an example showing the dichotomized risk scores from the original matrices (left) and the adjusted matrices (right) under a correlation threshold of 0.93 using the METABRIC data. Patient samples were separated into two groups according to the predicted risk scores from the selected genes. High-risk group is labelled in red and low-risk group is labelled in blue. The high-risk group is better separated from the low-risk group by using the MANCIE-adjusted expression data (right), compared with using the original data (left). (**b**) *P* value scores (−log_10_Pvalue) in survival prediction using METABRIC gene-expression data comparing before or after MANCIE adjustment with CNV data. The gene selection thresholds are set as 0.7, 0.75, 0.8, 0.85, 0.9, 0.93, from left to right, from top to bottom, respectively. (**c**) Difference of *P* value scores (−log_10_Pvalue) in survival prediction with each gene signature using TCGA gene-expression data before or after adjustment by MANCIE or SVA. Gene signatures are labelled with the first author name of the publication. Error bar stands for s.d. of the results from 1,000 random samples.

We applied MANCIE on the METABRIC breast cancer data sets^3^ to demonstrate its effectiveness for noise reduction on another data platform. METABRIC has two independent cohorts of breast cancer patients. Each cohort has around 1,000 patients with gene-expression values, CNV values and survival information. We used the first cohort as the training set and the second cohort as the independent set to predict survival information from gene-expression data. MANCIE was applied to adjust the gene-expression data based on CNV data. The underlying assumptions are: first, if gene-expression signatures can predict patient survival outcome, noise-reduced and bias-corrected gene-expression data should have better predictive accuracy in the patient survival; second, genes with concordant correlation between copy number and expression are more likely to be reliably measured and are more informative for outcome prediction. Indeed, we found that the MANCIE-adjusted data can better predict survival information than the original expression data, by better distinguishing patients with lower and higher risk of death, with an example shown in Fig. 3a. To assess the improvement quantitatively, we compared the logrank *P* values obtained using original training and original testing expression matrices with the logrank *P* values obtained using adjusted training and adjusted testing expression matrices. We limited our analysis to a subset of genes whose adjusted expression values are most different from the original values, defined as correlation of the adjusted vector and original vector being smaller than a threshold for either the training or the testing data set. Under a series of threshold from 0.7–0.93, MANCIE consistently improved the prediction accuracy by generating smaller *P* values (Fig. 3b).

Although TCGA also has breast cancer profiles, the death events are too few to provide meaningful survival separation. Therefore, we applied MANCIE on TCGA lung adenocarcinoma data^2^ for survival prediction. A total of 10,704 genes for 417 tumours with complete expression, CNV and clinical information were used, and MANCIE was applied to adjust the gene-expression data based on CNV data. For comparison, the gene-expression data matrix was also adjusted by the SVA method. To test the effectiveness of MANCIE adjustment, we selected six prognostic gene signatures for non-small cell lung cancer from previous publications^21^,^22^,^23^,^24^,^25^,^26^ for survival prediction. The 417 tumours were sub-sampled for 1,000 times when each time 90% of these samples were randomly selected. For each sub-sample, supervised principal components for survival analysis (SuperPC)^27^ was used to fit each gene signature and a continuous risk score is generated from the fitted model for the patients in the sub-sample. Then, similar to the METABRIC data analysis, a Cox proportional hazards model^28^ was regressed on the risk score to test how well the trained risk score can explain the survival data, with smaller *P* values indicating better correlation between the risk score and the survival data (one example shown in Supplementary Fig. 3a). We then plotted the differences in negative log *P* values before and after either MANCIE or SVA adjustment for each gene signature over the 1,000 samplings (Fig. 3c and Supplementary Fig. 3b). MANCIE adjustment can improve the *P* values, indicating better association of the risk scores with survival data for all six gene signatures (Fig. 3c), and outperformed SVA in four out of the six gene signatures.

In addition, we showed the effect of different combinations of the two cutoff parameters on the performance of MANCIE. We used the differential log *P* values to evaluate the improvement of survival prediction of MANCIE-adjusted data over the original data. For both METABRIC (Supplementary Fig. 4a) and TCGA lung cancer data (Supplementary Fig. 4b), although MANCIE improvement varies with the cutoffs, overall it is robust and has good performance under the default (0, 0.5) parameters.

### CCLE/GDSC data

We next demonstrated that MANCIE can reduce noise in a data set by leveraging information from a different source of the same data type. Two recent drug screen studies, CCLE^4^ and Genomics of Drug Sensitivity in Cancer (GDSC) project^29^ led by the Cancer Genome Project-profiled cancer cell line response to different drugs as well as the expression and CNV of each cell line. The two projects shared a total of 425 common cell lines and 10,461 genes measured. We adjusted the gene-expression data from CCLE project as the main matrix using the associated data matrix from the GDSC project, and compared the performance between MANCIE and SVA.

**Figure 4:**
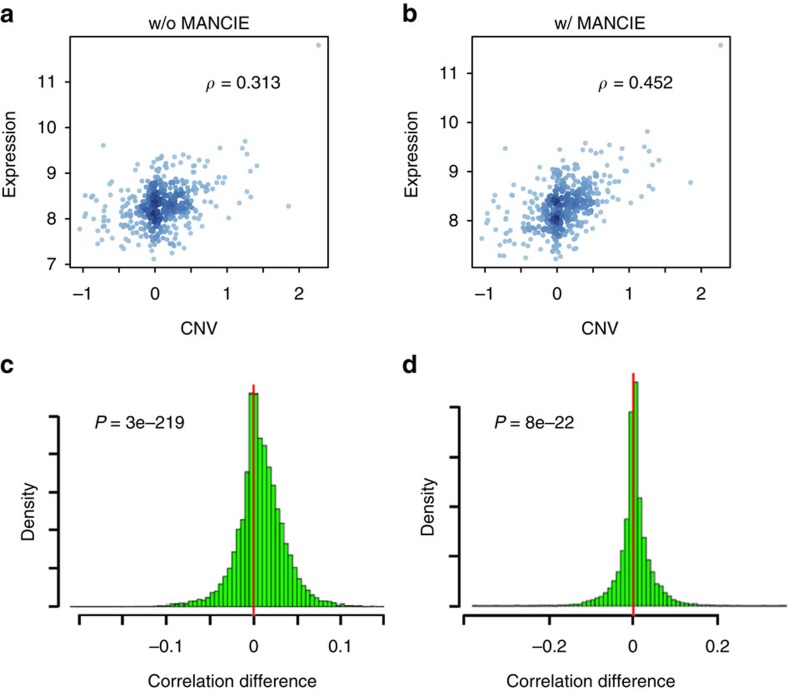
Case Study on CCLE/GDSC data. (**a**,**b**) Correlation between the CNV and RNA expression for gene *NDUFC2*. The expression data were using raw CCLE data (**a**) or MANCIE-adjusted with GDSC data (**b**). *ρ* refers to Spearman correlation coefficient. (**c**) Distribution of the correlation difference comparing before and after MANCIE adjustment, for all genes. The Spearman correlation coefficient between CNV and RNA expression was calculated for each gene, and the correlation difference is calculated by subtracting with MANCIE adjustment by without MANCIE adjustment. *P* value was calculated using the one-tail paired *t*-test. (**d**) Distribution of the correlation difference comparing comparing the raw data with SVA-adjusted expression data. *P* value was calculated using the one-tail paired *t*-test.

To evaluate the performance of MANCIE and SVA, the correlation between the expression level and CNV were examined for each gene using expression data with and without adjustment. The underlying assumption is that better noise reduction should give stronger correlation between expression and CNV as a general trend, given the fact that the expression of many genes is partially driven by its CNV. The difference in Spearman correlation after MANCIE adjustment is calculated for each gene (example of *NDUFC2* gene in Fig. 4a,b) and it is positive for 63% of the genes (Fig. 4c), compared with the 55% improved correlation with SVA (Fig. 4d). These results show that MANCIE is able to reduce noise in the expression data by using independent data from a different lab and to improve the overall data consistency.

## Discussion

We present a general computational method for high-dimensional genomic data integration. By case studies using available large-scale genomics data cohorts from several consortium efforts, we demonstrated that MANCIE can reduce potential biases and noises without identifying them in prior and successfully derive biologically meaningful information from data integration. As a data-driven method, MANCIE uses the basic principles from PCA by identifying the largest concordant differences across samples. MANCIE has few parameters, therefore making it more generally applicable to different data types. To our knowledge, MANCIE is the best-performing computational method for bias correction and integration of two high-dimensional genomic data matrices. In general, MANCIE can be applied to integrate any two data matrices with matched columns, For example, it can integrate genetic mutation profiles with gene-expression profiles, RNA-level expression with protein expression, or chromatin profiles generated using similar but different techniques, such as DNase-seq and ATAC-seq and so on.

Like every computational method for high-dimensional data analysis, MANCIE has its specific scope and limitation. It holds a strong assumption that the concordant information between the two data matrices is biologically meaningful. However, if the biases or batch effects exist in both the main and associated matrices in a concordant manner, MANCIE cannot identify them, and might even enhance the consistent bias with detrimental effects. In addition, MANCIE requires additional information, such as physical distance information, for the associated matrix summarization step. It assumes that, for one row in the main matrix, the principal direction of the related features in the associated matrix should contain concordance information that can be borrowed. For computational convenience, we usually only consider the local effect, that is, only include nearby associated data rows in the summarization of the associated matrix into a row that matches the main data row. There are situations when this might be insufficient, for example, enhancer regulation at distal loci could be ignored in the summarization step of MANCIE. Furthermore, MANCIE carries out the adjustment through each corresponding row vectors independently. It does not use the information across rows, which could be valuable and helpful in more sophisticated statistical inference to be explored in the near future. Last but not least, MANCIE prefers many columns in the data matrices, because more measured samples could potentially increase the power of bias modelling and correction.

We tested different combinations of cutoff values in the METABRIC and TCGA data. In these cases, previous prognostic signatures were used to evaluate and guide the choice of parameters, but this type of orthogonal information is not always available to optimize parameter selection. According to our experience with the example data sets in this study and for simplicity's sake, we set the default cutoff1 to be 0. The underlying assumption is that only positive correlations between the main row vector and the corresponding associated row vector are biologically meaningful and can be integrated. Cutoff2 can be empirically set so that about 1/3 of the rows are adjusted under the third scenario, which has worked robustly in all the example data sets in our study.

Taking together, MANCIE can be used as a general method for high-dimensional data integration with the potential to reduce noise and correct biases. It serves as a proof of principle that bias can be removed by such a methodology and more advanced statistical algorithm may be developed. For example, MANCIE can be extended to include multiple associated matrices for correction of bias in the one main matrix. It provides a novel approach to genomic data integration, an area that will become increasingly important for mining big biological data in the near future.

## Methods

### MANCIE algorithm

*Data description*. The main matrix is denoted as 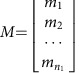. The main matrix can be any high-dimensional genomic data such as gene expression or peak count data across many different samples/conditions (listed on columns). The main matrix can come from high-throughput experiments such as RNA-Seq, microarray, ChIP-Seq, DNase-Seq, ATAC-seq and so on. Each row vector *m*_*i*_,*i*=1, 2, … *n*_1_ represents a feature such as one gene or one peak. Similarly, an associated matrix is defined as 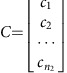, where each row vector *c*_*i*_,*i*=1, 2, … *n*_2_ represents a feature such as one gene or one peak. The associated matrix *C* could come from the same or different type of experiment as *M*, but must be conducted in exactly the same sample conditions as *M*. In other word, *M* and *C* must have matched columns. *n*_1_=*n*_2_ is satisfied when *M* and *C* are from the same experiment and may not be satisfied when *M* and *C* are from different experiments. In the latter case, one matrix could be DNAase-Seq and the other matrix could be RNA-Seq. For such scenarios, the annotation data relating both the main and associated matrix (for example, chromosome, start position of feature and ending position of feature) must be available.

*Summarization of information in the associated matrix*. This step is skipped when each row vector in *C* has a one-to-one matching row vector in *M*, which measures different features of the same entity. For example, when a gene's expression is measured in *M* and the same gene's CNV data is measured in *C*, the *M* and *C* matrices satisfy this condition. Otherwise, the *C* data need to be summarized to this format that is compatible with the *M* matrix in both dimensionality and biological meaning.

On the basis of the connection information, for each row in the main matrix, no more than *p* rows in the associated matrix associated with the row in the main matrix were selected and the first principal component of this sub-matrix is calculated as the summarized row in the summarized associated matrix (Supplementary Fig. 1).

One summarization function that works in the majority of all cases is described here. For each row vector in *M*, *m*_*i*_, the nearest features defined by physical genomic distances calculated in the annotation data in the *C* matrix are extracted. The number of nearest features to extract can be set flexibly by users. Let 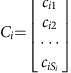
be the subset of *C* that are extracted whose features are closest to *m*_*i*_. If not a single feature in *C* are found to be close to *m*_*i*_ in the main matrix, this main matrix row will be skipped for adjustment. Then the first principal component of 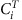 by PCA on correlation matrix is calculated as a row vector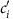. In the case of Pearson correlation 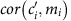 smaller than 0, all elements in 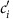 are converted to their additive inverse. These summarized vectors form a new matrix 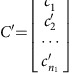. The new matrix *C*′ is compatible in dimension with *M* and each row represents summarized information carried by *C* that might be leveraged to remove the noise in the corresponding row in *M*.

*Removing noise in *M* matrix by combination with *C**. With slight abuse of terms, if step (2) is carried out, still let *C* denote the new matrix *C*′. Now for each row *m*_*i*_, a three-way strategy is employed to remove noise by borrowing information from the associated matrix *C*.

(a) If *cor*(*c*_*i*_, *m*_*i*_)<=*cutoff*1, the new row vector 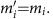


(b) If *cor*(*c*_*i*_, *m*_*i*_)>*cutoff*1 and *cor*(*c*_*i*_, *m*_*i*_)<=*cutoff*2, the new row vector 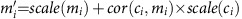, where *cor*(*c*_*i*_, *m*_*i*_) is the Pearson correlation and *scale*(*x*) is a function that scales a row vector to an s.d. of 1.

(c) If *cor*(*c*_*i*_, *m*_*i*_)>*cutoff*2, the new row vector 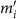 is the first principal component of the PCA of 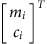 on the 2 × 2 correlation matrix. In the case of Pearson correlation 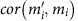 smaller than 0, all elements in 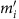 are converted to their additive inverse.

The two cutoffs *cutoff*1 and *cutoff*2 can be set flexibly by the users, with default values of 0 and 0.5. In case summarization is involved and if the first cutoff is set to be equal or less than 0, the first option will not be used. Finally, 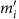 calculated by either of the three options is scaled to have the mean and s.d. of the old row vector *m*_*i*_. The adjusted main matrix is then 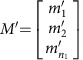.

### ENCODE data analysis

DNase-Seq data sets from each individual cell line was downloaded from ENCODE^1^. DHS peaks were identified in each cell line using MACS2^30^ with default parameters and only peaks whose summits are in introns or intergenic regions and have a fold enrichment of at least four were retained. Peaks wider than 150 bp were chopped to 150 bp centred at the summit location. Then overlapping DHS peaks were merged into a new region. The DHS level on each merged peak in each cell line were measured as RPKM. Exon array data were processed using JETTA^31^. The sequence motif scan analyses were performed using the MDSeqPos algorithm^32^ on the Cistrome analysis pipeline^33^.

### Survival analysis for METABRIC data set

We adjusted both the training set expression matrix and the testing set expression matrix with the corresponding CNV data matrix using default parameters. Then we calculate the Pearson correlation of each row vector in the original training set expression matrix and the row vector in the adjusted training set expression matrix. We also calculated the Pearson correlation for the testing set matrix before and after adjustment. We focus our analysis on the row vectors (gene expression across patients) whose Pearson correlations are below a certain threshold for either the training set or the testing set. To be objective, we chose a series of threshold values in the downstream analysis. Then we used LASSO^34^ with cox family to analyse selected genes from the previous step to further narrow down the genes whose expression levels are significantly correlated with survival in the training set. Then we fit a multivariate Coxph model^35^ using these selected genes and predicted to the testing set after which Coxph model returned a risk score vector for the testing set patients. We dichotomized the risk score based on median risk and tested the Logrank *P* values of the overall survival difference between the low-risk group and high-risk group. Since the LASSO method is stochastic, generating slightly different results especially when the number of input features is large, we ran the same analysis 20 times and obtained a distribution of logrank *P* values.

### Data analysis by SVA

For TCGA data, we used cancer stage and smoking status as the primary variables, no variable of known noise source and 1 surrogate variable to be estimated. For CCLE/GDSC data, we used no variable of known noise source and 1 surrogate variable to be estimated. For Encode data, we used tissue type as known noise source and 1 surrogate variable to be estimated. Each row vector is regressed on the estimated surrogate variable and replaced by the regression residuals.

### Choice of parameters

For METABRIC and TCGA data studied in this paper, Cutoff1 was set as 0 and the Cutoff2 being 0.5. For CCLE data, the two parameters are 0 and 0.7, respectively. For Encode data, the two parameters are 0 and 0.5, respectively. We used the differences in negative log rank *P* values as a metric to evaluate the effect of different combinations of higher cutoff and lower cutoff on the efficiency of MANCIE for the METABRIC data set.

### Availability

MANCIE is available as an R-package (http://cran.r-project.org/web/packages/MANCIE/).

## Additional information

**How to cite this article:** Zang, C. *et al*. High-dimensional genomic data bias correction and data integration using MANCIE. *Nat. Commun.* 7:11305 doi: 10.1038/ncomms11305 (2016).

## Supplementary Material

Supplementary Information Supplementary Figures 1-4, Supplementary Note 1.
