# Supplementary material for: High-dimensional genomic data bias correction and data integration using MANCIE
Source: Nat Commun. 2016 Apr 13;7:11305. doi: 10.1038/ncomms11305 (PMC4833864; doi:10.1038/ncomms11305)
Supplement: Supplementary Information — Supplementary Figures 1-4, Supplementary Note 1. [file ncomms11305-s1.pdf]

Supplementary Figure 1

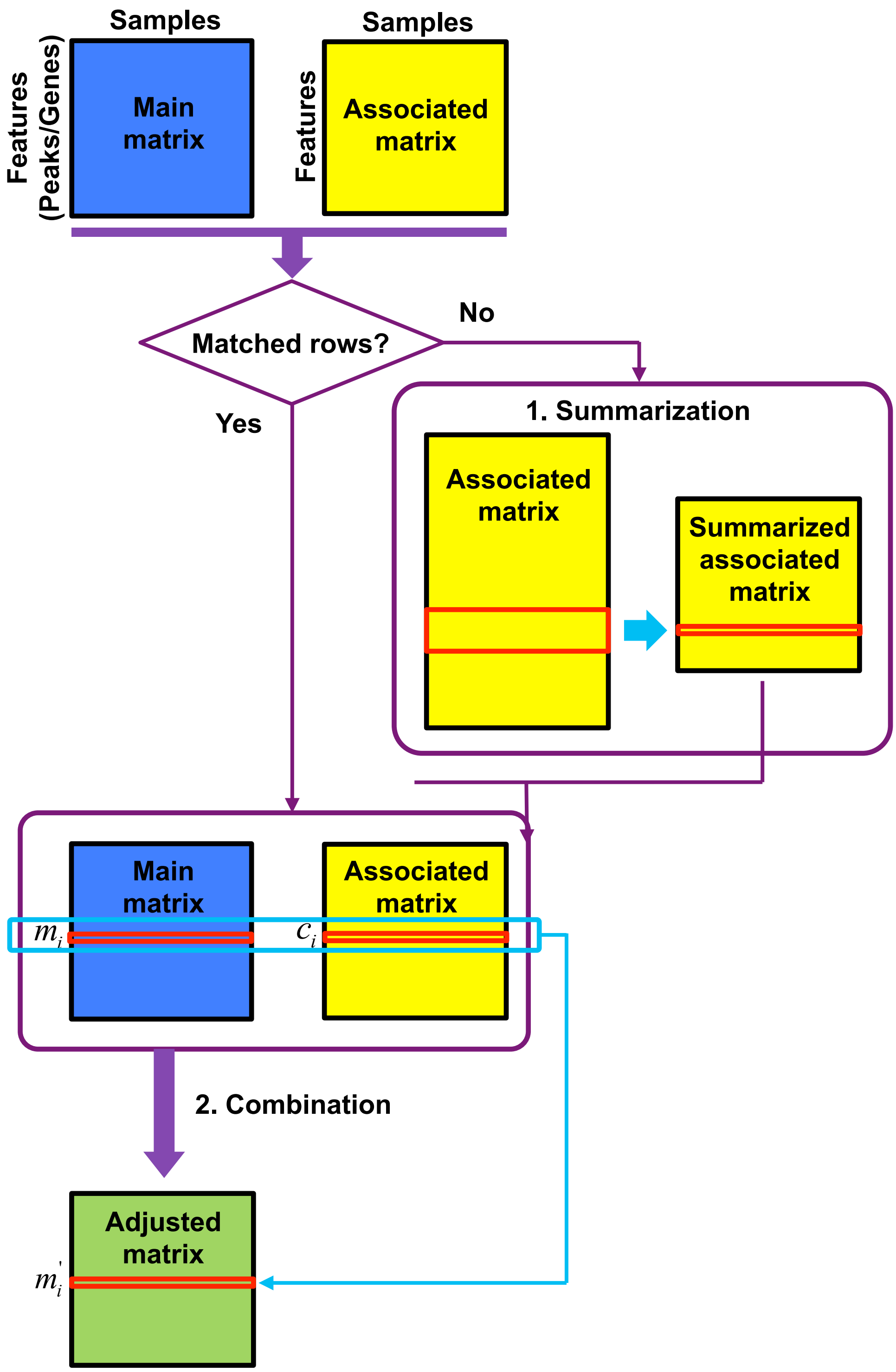

Supplementary Figure 1. MANCIE workflow. If the rows in the associated matrix and the main matrix do not match, the summarization step converts the associated matrix to a summarized associated matrix with matched rows. The combination step integrates the main matrix with the summarized associated matrix into the adjusted matrix.

Supplementary Figure 2

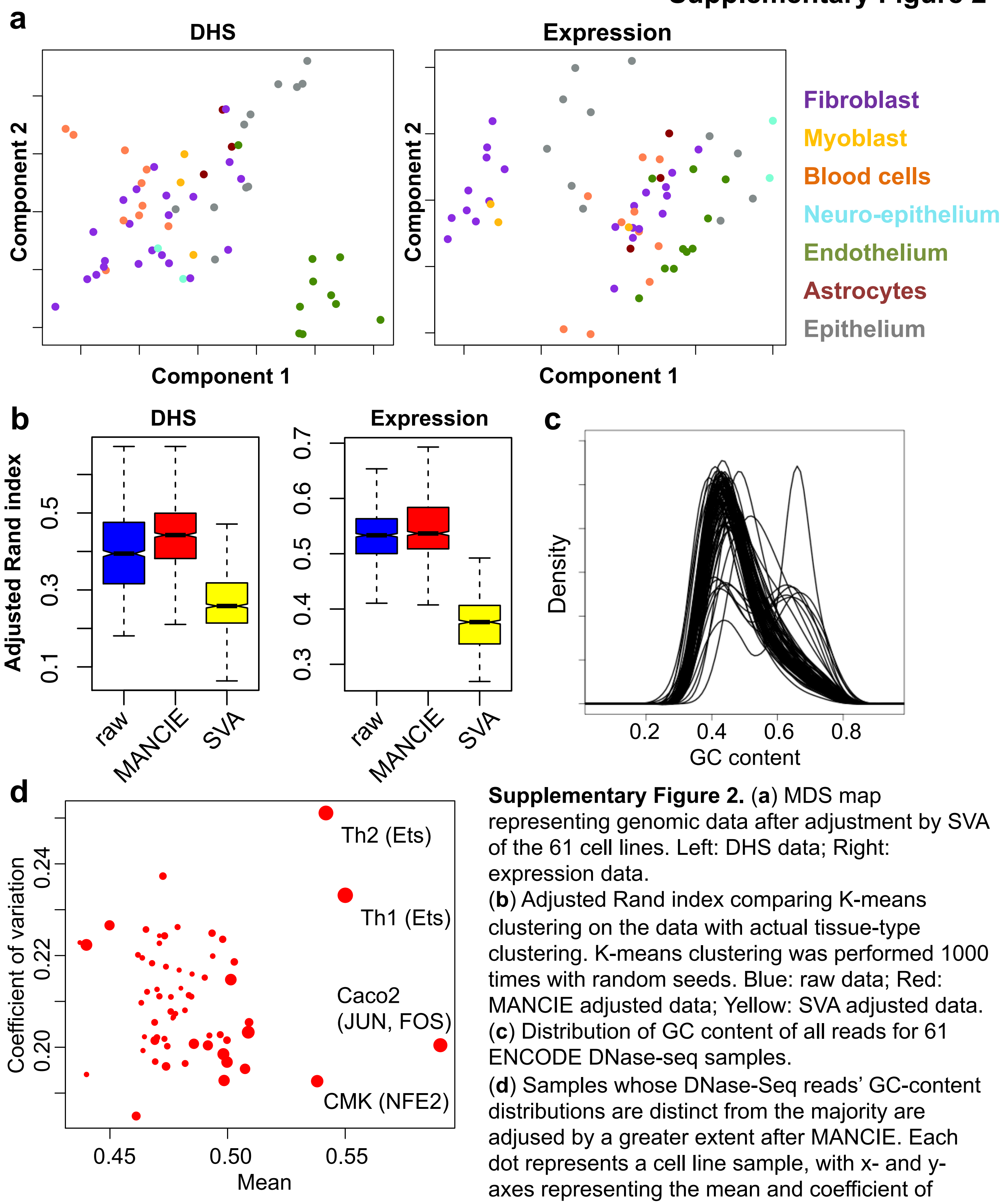

Supplementary Figure 2. (**a**) MDS map representing genomic data after adjustment by SVA of the 61 cell lines. Left: DHS data; Right: expression data.
(**b**) Adjusted Rand index comparing K-means clustering on the data with actual tissue-type clustering. K-means clustering was performed 1000 times with random seeds. Blue: raw data; Red: MANCIE adjusted data; Yellow: SVA adjusted data.
(**c**) Distribution of GC content of all reads for 61 ENCODE DNase-seq samples.
(**d**) Samples whose DNase-Seq reads' GC-content distributions are distinct from the majority are adjused by a greater extent after MANCIE. Each dot represents a cell line sample, with x- and y-axes representing the mean and coefficient of variation, respectively, of the CG-content distribution of all reads in the DNase-seq dataset. The size of the dot represents the magnitude of adjustment of MANCIE, measured by the Euclidean distance between the sample data vectors before and after MANCIE adjustment.

Supplementary Figure 3

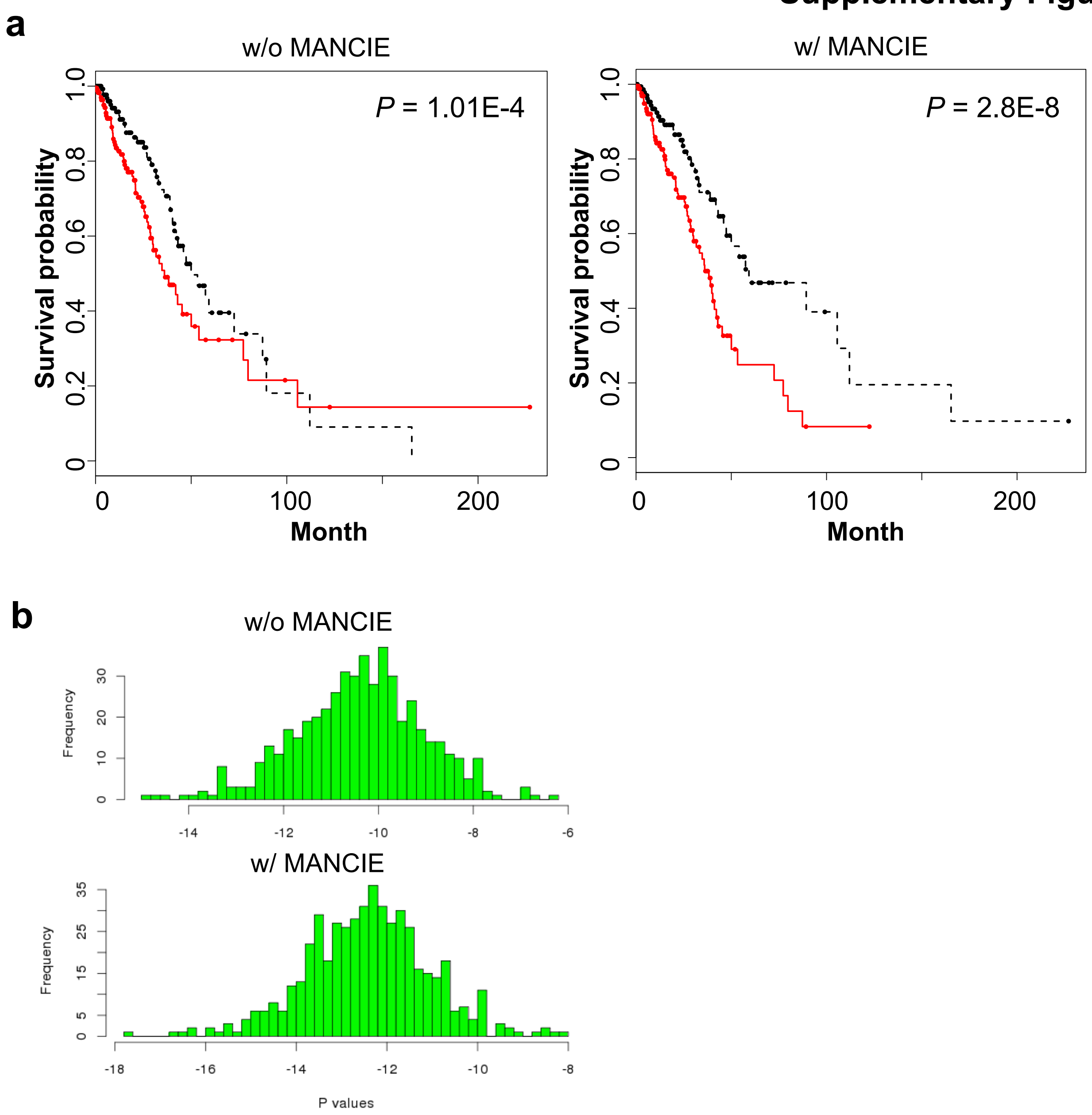

Supplementary Figure 3. (**a**) The Kaplan-Meier plots for an example showing differences in survival prediction accuracy and the improvement in P-value, using the Beer gene signature. Patient samples were separated into two groups according to the trained risk score using the gene signature with the expression data. High-risk group is labeled in red and low-risk group is labeled in black. The high-risk group is better separated from the low-risk group by using the MANCIE-adjusted expression data (right), compared with using the raw data (left).

(**b**) Distribution of P-value scores (-log10Pvalue) for the prognostic prediction with the Beer gene signature, comparing raw expression data (top) and MANCIE-adjusted expression data (bottom), from TCGA lung cancer (LUAD) cohort.

Supplementary Figure 4

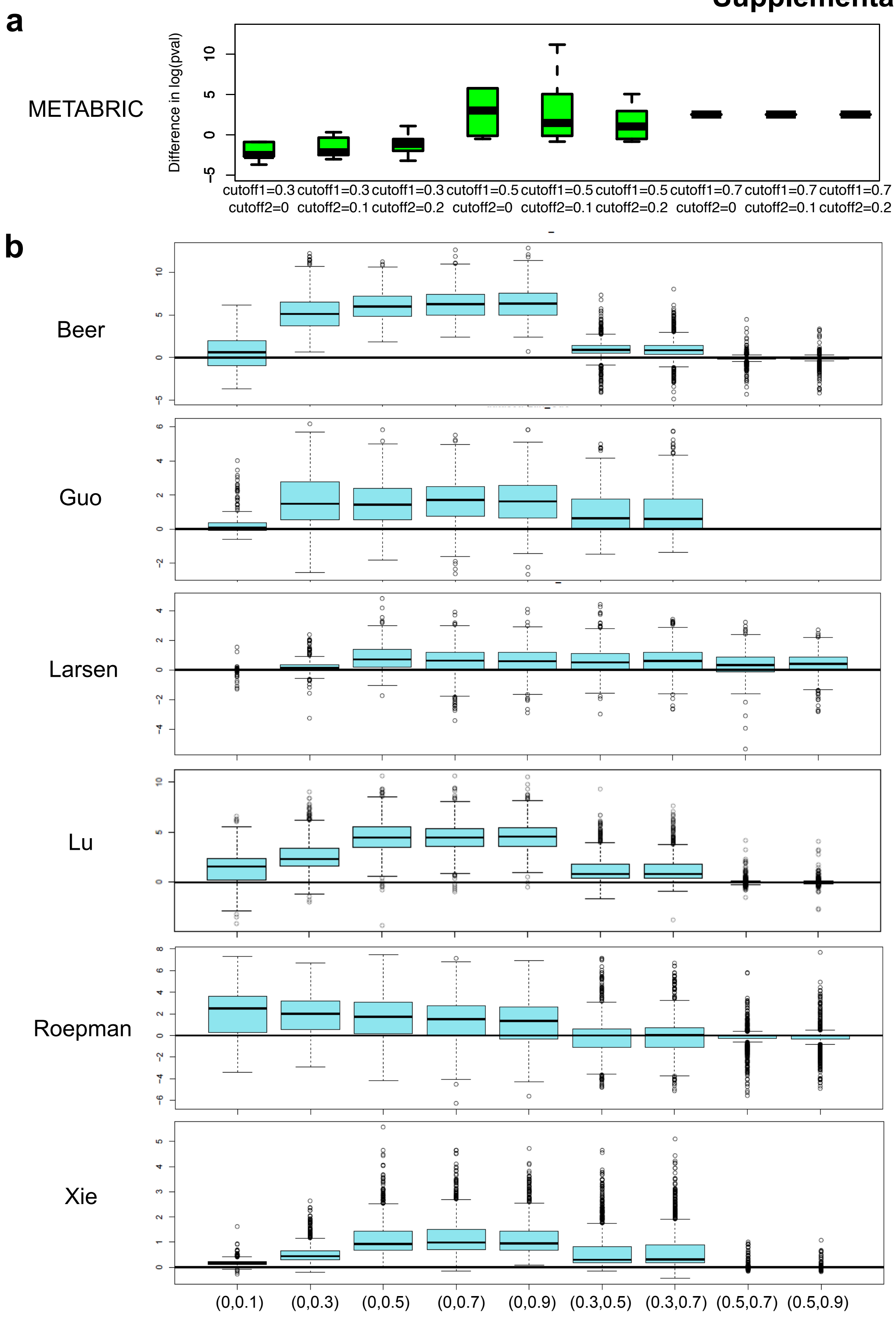

Supplementary Figure 4. P-value improvement on the METABRIC data (**a**) and the 6 gene signatures from TCGA data (**b**) under combinations of different MANCIE parameters. Y-axis indicates the difference of P-value scores similar to **Fig. 3c**. Each box plot represents a parameter setting, labeled as (cutoff1, cutoff2) at the bottom.

Supplementary Note 1

Theoretical Support for MANCIE: an Approximation of Rigorous Statistical Inference

Problem Description. Let $m_i = (m_{i1}, \cdots, m_{iK})$ be the i-th row (i.e., feature) of the main matrix M, and $c_i = (c_{i1}, \cdots, c_{iK})$ be its counterpart in the associated matrix C, where each $k \in \{1, \cdots, K\}$ stands for one sample or condition. Since $(m_{ik}, c_{ik})^T$ are observations of feature i from different biological experiments which often contain a lot of uncertainty, it's natural to assume that they are the noised version of the underlying "truth" $(m^*_{ik}, c^*_{ik})^T$, i.e.,

$$(m_{ik}, c_{ik})^T = (m^*_{ik}, c^*_{ik})^T + \varepsilon_{ik},$$

where ε_{ik} is a two-dimensional noise vector. MANCIE aims to remove noise in m_i by borrowing information from c_i, i.e., inferring $m^*_i = (m^*_{i1}, \cdots, m^*_{iK})$ based on both m_i and c_i.

Statistical Model & Inference. To simplify the problem, let's assume that both $\{(m^*_{ik}, c^*_{ik})^T\}_{k=1}^K$ and $\{\varepsilon_{ik}\}_{k=1}^K$ are i.i.d. samples of Gaussian distributions, i.e.,

$$(m^*_{ik}, c^*_{ik})^T \sim N\big(\mu_i, \Sigma_i\big) \quad \text{and} \quad \varepsilon_{ik} \sim N\big(\mathbf{0}, \Delta_i\big),$$

where $\Sigma_i = \begin{pmatrix} 1 & \rho_i \\ \rho_i & 1 \end{pmatrix}$, $\Delta_i = \begin{pmatrix} \delta^2_{im} & 0 \\ 0 & \delta^2_{ic} \end{pmatrix}$, $\{(m^*_{ik}, c^*_{ik})^T\}_{k=1}^K$ and $\{\varepsilon_{ik}\}_{k=1}^K$ are independent of each other, and $\rho_i > 0$. Clearly, δ^2_{im} and δ^2_{ic} stands for the noise-signal ratio of m_i and c_i respectively, where a larger δ^2 means lower quality of the data. Here, we assume that $\delta^2_{ic} \geq \delta^2_{im}$ as the main matrix usually enjoys better quality.

Under this model, we have

$$\begin{aligned} (m_{ik}, c_{ik})^T \mid (m^*_{ik}, c^*_{ik})^T &\sim N\big((m^*_{ik}, c^*_{ik})^T, \Delta_i\big), \\ (m_{ik}, c_{ik})^T &\sim N\big(\mu_i, \Sigma_i + \Delta_i\big). \end{aligned}$$

And, it's easy to check that

$$\tilde{\rho}_i = cor(m_i, c_i) = \frac{\rho_i}{\sqrt{(1+\delta^2_{im})(1+\delta^2_{ic})}} \leq \rho_i = cor(m^*_i, c^*_i),$$

i.e., the correlation coefficient of the observed data (m_i, c_i) is always smaller than the true correlation coefficient $cov(c^*_i, m^*_i)$, and the difference depends on the noise level $(\delta^2_{im}, \delta^2_{ic})$.

Without loss of generality, we can also assume that $\mu_i = \mathbf{0}$ for any feature i (i.e., the observed data are centralized).

Now, assume that both Σ_i and Δ_i are known. Based on the Bayes rule, we have the following posterior distribution for $(m_{ik}^*, c_{ik}^*)^T$:

$$\begin{aligned}
& f\big((m_{ik}^*, c_{ik}^*)^T \mid (m_{ik}, c_{ik})^T\big) \\
\propto\ & \pi\big((m_{ik}^*, c_{ik}^*)^T\big) \cdot f\big((m_{ik}, c_{ik})^T \mid (m_{ik}^*, c_{ik}^*)^T\big) \\
\propto\ & \exp\Big\{-\frac{1}{2}(m_{ik}^*, c_{ik}^*)\Sigma_i^{-1}(m_{ik}^*, c_{ik}^*)^T\Big\} \times \\
& \exp\Big\{-\frac{1}{2}\big[(m_{ik}, c_{ik}) - (m_{ik}^*, c_{ik}^*)\big]\Delta_i^{-1}\big[(m_{ik}, c_{ik}) - (m_{ik}^*, c_{ik}^*)\big]^T\Big\} \\
\sim\ & N\big((\Sigma_i^{-1} + \Delta_i^{-1})^{-1}\Delta_i^{-1}(m_{ik}, c_{ik})^T, (\Sigma_i^{-1} + \Delta_i^{-1})^{-1}\big),
\end{aligned}$$

which means that the best guess for the unknown $(m_{ik}^*, c_{ik}^*)^T$ should be the posterior mean

$$\begin{aligned}
\nu_{ik} &= (\Sigma_i^{-1} + \Delta_i^{-1})^{-1}\Delta_i^{-1}(m_{ik}, c_{ik})^T \\
&\propto \begin{pmatrix} \delta_{ic}^2 + 1 - \rho_i^2 & \rho_i\delta_{im}^2 \\ \rho_i\delta_{ic}^2 & \delta_{im}^2 + 1 - \rho_i^2 \end{pmatrix} \begin{pmatrix} m_{ik} \\ c_{ik} \end{pmatrix}.
\end{aligned}$$

Since we are interested in improving the main matrix, we will only focus on

$$E(m_{ik}^* \mid m_{ik}, c_{ik}) \ \propto\ m_{ik} + \frac{\rho_i\delta_{im}^2}{\delta_{ic}^2 + (1-\rho_i^2)} \cdot c_{ik}.$$

MANCIE as an Approximation of Rigorous Statistical Inference. In practice, however, we can only estimate $(\Sigma_i + \Delta_i)$ from the observed data m_i and c_i, and neither Σ_i or Δ_i is estimable on their own. Therefore, $h(\rho_i) = \frac{\rho_i\delta_{im}^2}{\delta_{ic}^2+(1-\rho_i^2)}$, and thus $E(m_{ik}^* \mid m_{ik}, c_{ik})$, cannot be precisely known. Fortuanately, the following facts hold for $h(\rho_i)$:

$$\begin{aligned}
(F_1) \qquad & h(\rho_i) \approx 0 \text{ if } \rho_i \text{ is very close to } 0, \\
(F_2) \qquad & h(\rho_i) \approx \frac{\rho_i\delta_{im}^2}{\delta_{ic}^2 + 1} \text{ if } \rho_i \text{ is close to } 0, \\
(F_3) \qquad & h(\rho_i) \approx \frac{\delta_{im}^2}{\delta_{ic}^2} \text{ if } \rho_i \text{ is very close to } 1,
\end{aligned}$$

which correspond to the three scenarios (a), (b) and (c) in the subsection of "Removing noise in matrix" respectively under proper conditions.

Clearly, (F_1) matches to scenarios (a). Let $\tilde{m}_i = \frac{m_i}{\sqrt{\delta_{im}^2+1}}$ and $\tilde{c}_i = \frac{c_i}{\sqrt{\delta_{ic}^2+1}}$ be the rescaled data (i.e., "scale(m_i)" and "scale(c_i)" in the paper). The new vector m' defined in scenarios (b) is:

$$m'_i = \tilde{m}_i + \tilde{\rho}_i \cdot \tilde{c}_i = \frac{m_i}{\sqrt{\delta_{im}^2+1}} + \frac{\rho_i}{\sqrt{(\delta_{im}^2+1)(\delta_{ic}^2+1)}} \cdot \frac{c_i}{\sqrt{\delta_{ic}^2+1}} \propto m_i + \frac{\rho_i}{\delta_{ic}^2+1} \cdot c_i,$$

which matches to (F_2) when δ_{im}^2 is close to 1. For scenarios (c), because $cor(m_i, c_i) = \Sigma_i + \Delta_i$, the first principle component of $cor(m_i, c_i)$ is $(1, \frac{r_i+\sqrt{r_i^2+4}}{2})$, where $r_i = \frac{\delta_{ic}^2-\delta_{im}^2}{\rho_i}$. Thus, the new vector m' defined in this scenario is

$$m'_i \propto m_i + \frac{r_i + \sqrt{r_i^2+4}}{2} c_i,$$

which degenerates to $(m_i + c_i)$ when r_i is close to 0 (this happens when $\delta_{im}^2 \approx \delta_{ic}^2$ and the difference $\delta_{ic}^2 - \delta_{im}^2$ is small compared to ρ_i). Considering that $h(\rho_i)$ also degenerates to 1 when $\delta_{im}^2 \approx \delta_{ic}^2$ in (F_3), we find that (F_3) matches to scenarios (c) when δ_{im}^2 and δ_{ic}^2 are close to each other. Summarizing all three cases, we conclude that MANCIE is a proper approximation of rigorous statistical inference when the noise-signal ratio in both the main matrix and association matrix are close to 1.
